# Supplementary figures and images for: Field assessment of BinaxNOW antigen tests as COVID-19 treatment entry point at a community testing site in San Francisco during evolving omicron surges
Source: PLoS One. 2023 Mar 24;18(3):e0283576. doi: 10.1371/journal.pone.0283576 (PMC10038282; doi:10.1371/journal.pone.0283576)

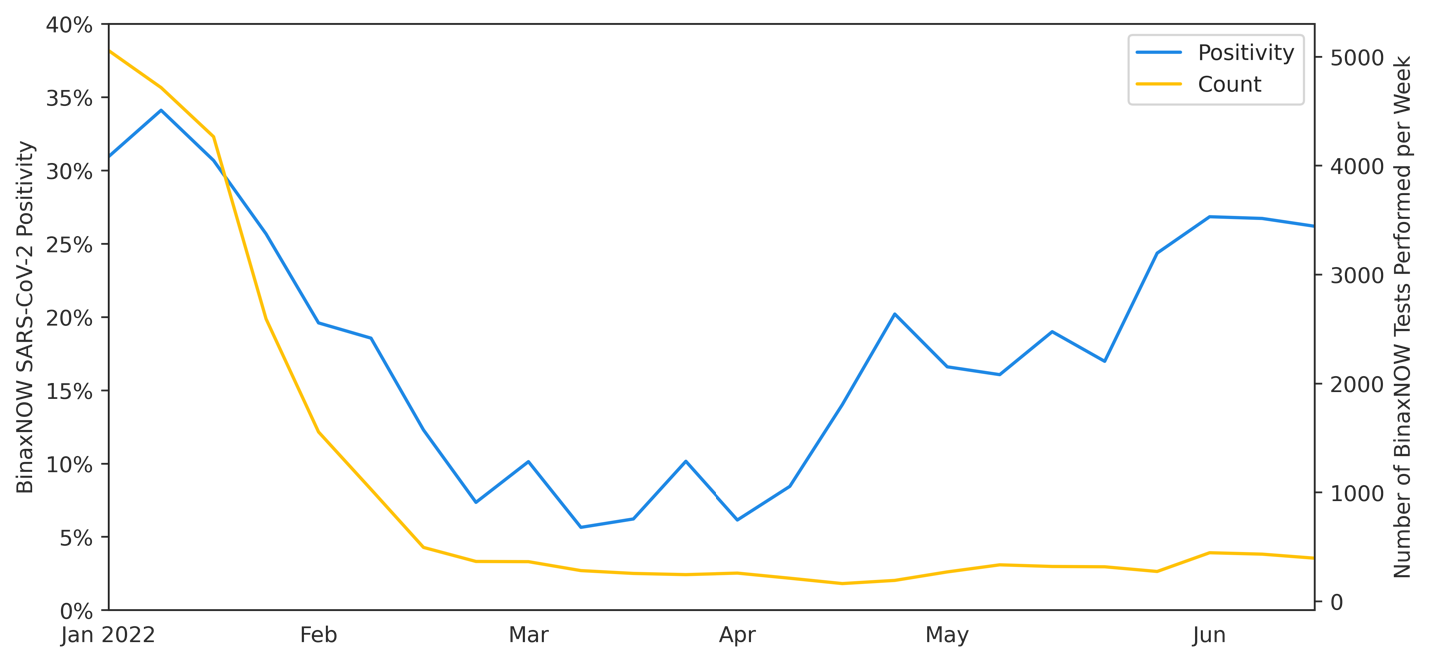

Supplement: S1 Fig — The blue line indicates the test positivity over time, corresponding with the left axis. The yellow line indicates the number of tests performed weekly, corresponding with the right axis. (TIF) [file pone.0283576.s001.tif]

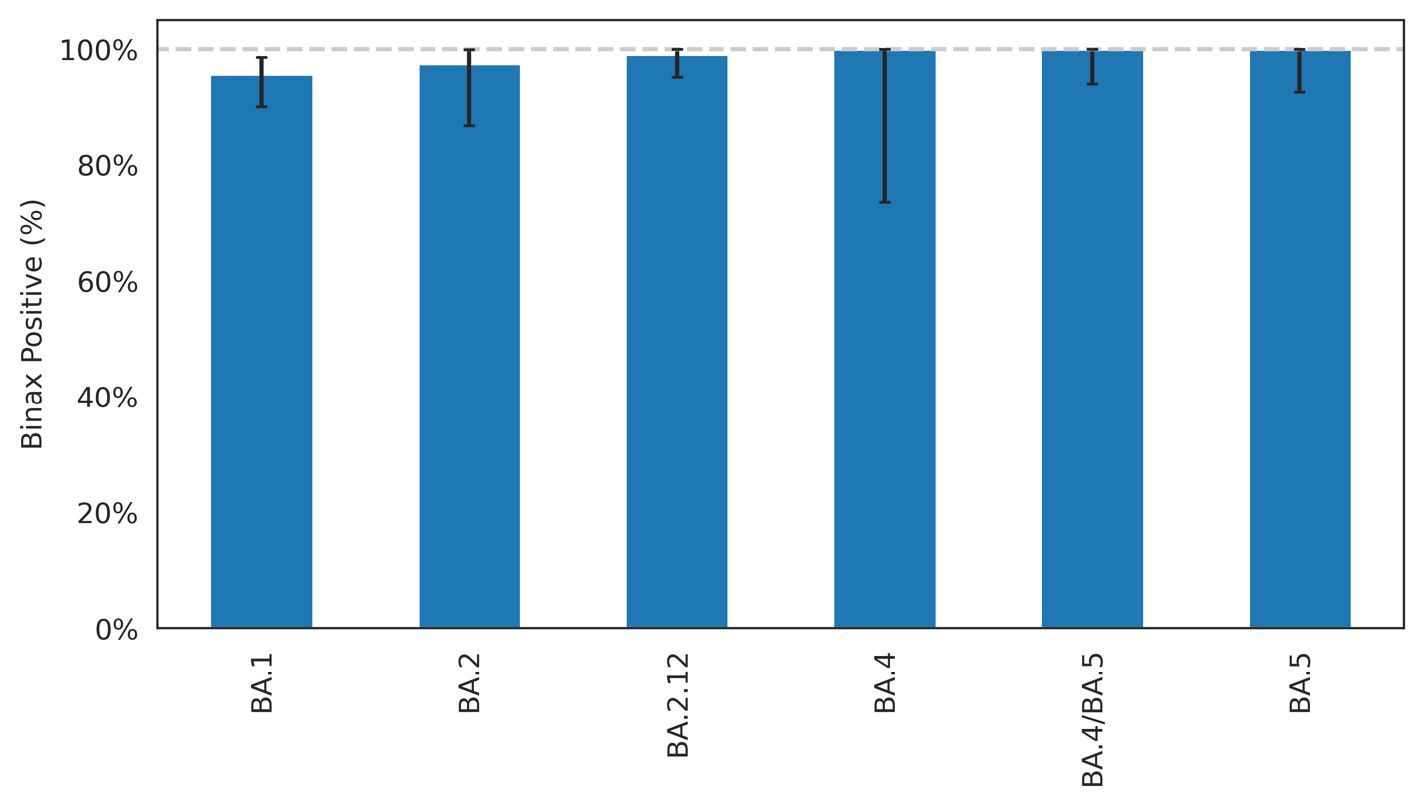

Supplement: S2 Fig — Each bar represents the BinaxNOW positivity rate among the corresponding lineage and related sublineages, with 95% confidence intervals. The grey dashed line corresponds with 100% of SARS-CoV-2 RT-PCR positive participants with Ct < 30 also testing positive on BinaxNOW. (TIF) [file pone.0283576.s002.tif]

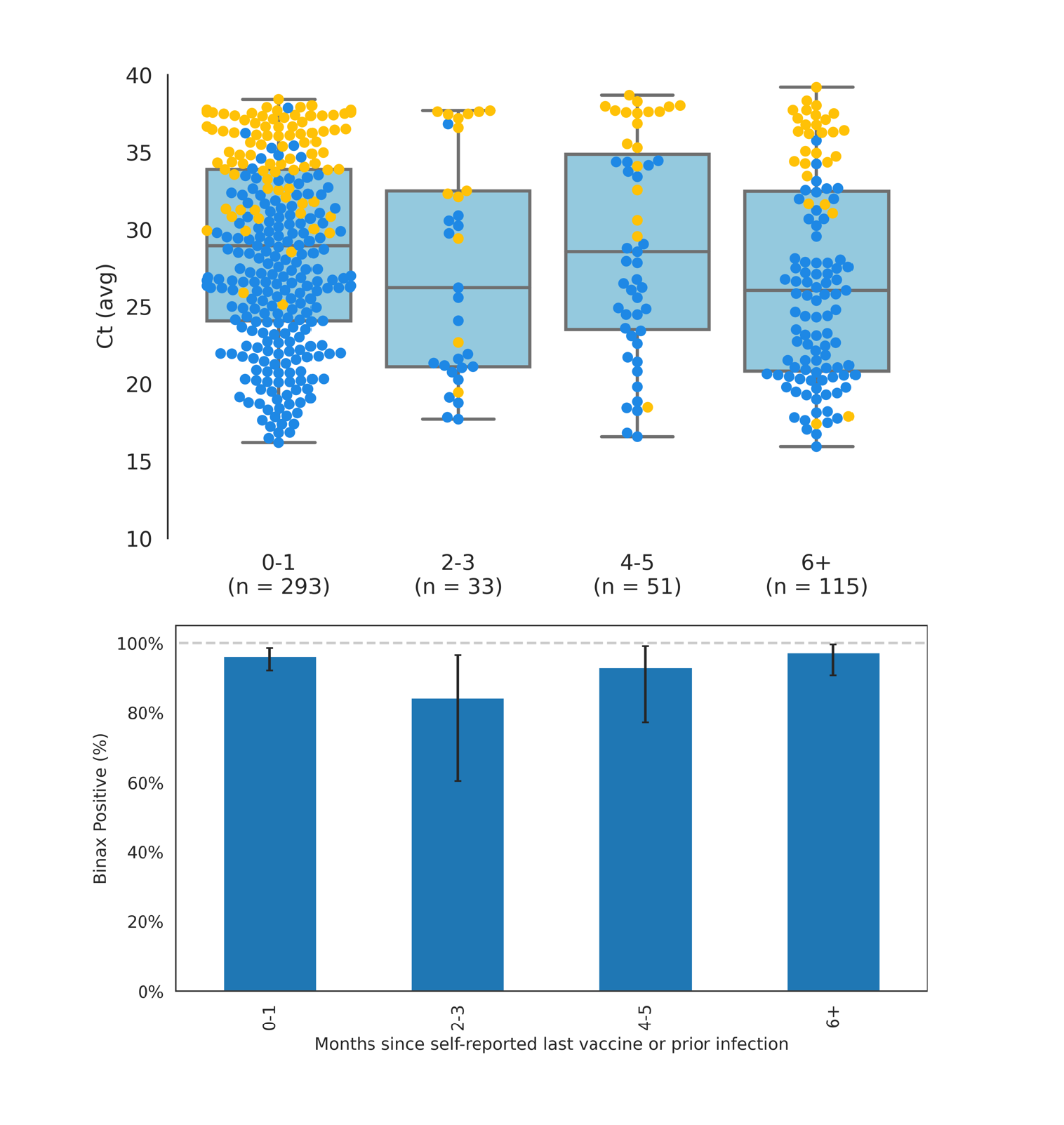

Supplement: S3 Fig — SARS-CoV-2 RT-PCR Ct values and BinaxNOW rapid antigen test results (panel a), and BinaxNOW rapid antigen test detection (panel b) for participants tested between January 1 and June 26, 2022, stratified by months since prior self-reported vaccine or infection status. Panel A shows box plot shows first quartile, median, and third quartile in the shaded region; diamonds indicate outliers beyond 1.5 times the interquartile range. Blue circles overlaid are individuals whose samples were positive on both rapid antigen test (BinaxNOW) and on RT-PCR test. Orange circles represent individuals who were RT-PCR positive but rapid antigen test negative. Panel B shows bar plots with percent of RT-PCR positive participants with SARS-CoV-2 RT-PCR Ct less than 30 who were also BinaxNOW positive. Error bars indicate 95% confidence interval for each estimate. The grey dashed line corresponds with 100% of SARS-CoV-2 RT-PCR positive participants with Ct < 30 also testing positive on BinaxNOW. Abbreviations: Ct, cycle threshold; RT-PCR, reverse transcriptase polymerase chain reaction. (TIF) [file pone.0283576.s003.tif]

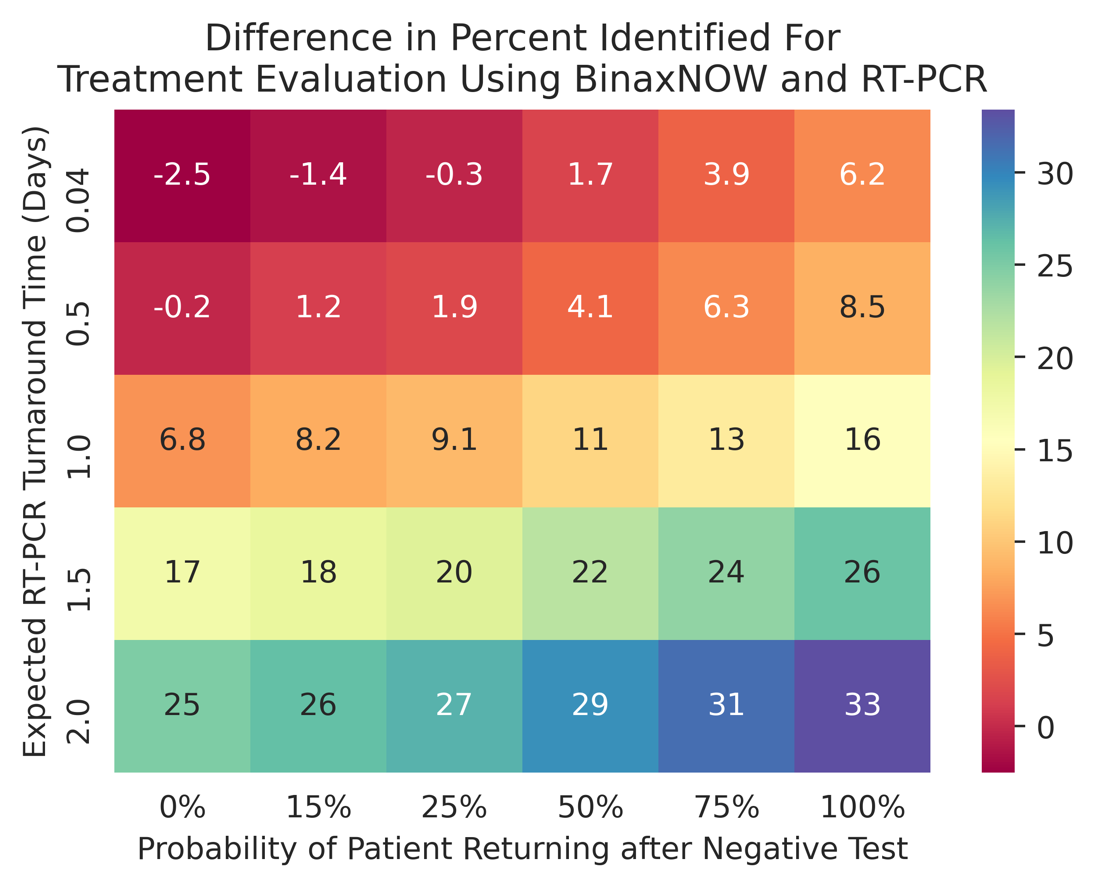

Supplement: S4 Fig — Rows correspond to variations of the expected time to receive RT-PCR results in days, as modeled by an exponentially distributed random variable; the rows correspond with one hour, half day, full day, day and a half, or two days. The columns correspond with the expected percent of patients returning and testing positive after a false negative, as modeled by a binomially distributed random variable; the columns correspond with 0%, 15%, 25%, 50%, 75%, and 100%. Each cell corresponds with the expected difference between the percent of participants being eligible for oral treatment based on rapid antigen test minus those eligible based on RT-PCR; a positive number indicates rapid antigen test identifying more participants, and a negative number indicated RT-PCR identifying more. Abbreviations: Ct, cycle threshold; RT-PCR, reverse transcriptase polymerase chain reaction. (TIF) [file pone.0283576.s004.tif]
