# Supplementary material for: Field assessment of BinaxNOW antigen tests as COVID-19 treatment entry point at a community testing site in San Francisco during evolving omicron surges
Source: PLoS One. 2023 Mar 24;18(3):e0283576. doi: 10.1371/journal.pone.0283576 (PMC10038282; doi:10.1371/journal.pone.0283576)
Supplement: Attachment — Submitted filename: Rebuttal Letter - PLoS ttt - Schrom et al.pdf [file pone.0283576.s005.pdf]

Division of HIV, Infectious Diseases & Global Medicine
Department of Medicine

ZUCKERBERG SAN FRANCISCO GENERAL
Hospital and Trauma Center

995 Potrero Ave,
Building 80, Ward 84,
San Francisco, CA 94110

Dear PLoS ONE Editors and Reviewers,

We appreciate you for taking the time in reviewing our paper and providing valuable comments. The authors carefully considered the comments and have addressed them below in italic type and in our revised manuscript.

We hope the revised version is now suitable for publication and look forward to hearing from you.

Sincerely,

Diane V. Havlir

Diane V. Havlir, MD
Professor and Associate Chair of Clinical Research, Department of Medicine
Chief, Division of HIV, Infectious Diseases and Global Medicine
Director, AIDS Research Institute
Robert L. Weiss Memorial Chair for HIV/AIDS Research
University of California, San Francisco | Zuckerberg San Francisco General Hospital

Reviewer Comments to the Author:

Reviewer #1: In this manuscript, Schrom et al performed simultaneous BinaxNOW and PCR testing on individuals presenting for testing at a community site in San Francisco. An impressive number of tests were performed- 25,309, with 2799 simultaneously being tested by PCR; sequencing demonstrated that the strains tested represented 5 omicron subvariants in circulation this year. To my reading, the manuscript meets the criteria for publication at PLoS One; changing framing somewhat could improve the readability and potentially the impact of the paper.

The primary knowledge gap that the manuscript is seeking to address is not clear. In some places, it sounds like the manuscript is looking to ask whether RATs (specifically BinaxNOW) can be useful as initial tests for treatment initiation from a timing standpoint. In other places it sounds like the manuscript is looking to ask whether BinaxNOW can readily detect the omicron variants that have been in circulation this year. Clinical practice in COVID-19 has changed rapidly, and in many (if not most) places, home RATs are the test that most commonly leads to treatment initiation. The framing of the manuscript has not been changed to accommodate this reality, leaving the reader to wonder why the work was performed. Some reframing to acknowledge this practical reality and contextualize the results within it would substantially improve the manuscript.

Response to Reviewer #1 Comments:

We appreciate the reviewer's comment and have updated the framing of the manuscript to address contemporary issues in the field. We agree with the reviewer that availability of home tests has changed the landscape of entry into treatment for many persons. However, low-income community members still have limited access to over-the-counter tests because of their high cost; in addition, many high-risk individuals lack agency to obtain free tests, and the allotted number can be insufficient for large households. Finally, rigorous data using specific rapid tests for detection of the evolving variants are lacking; in the absence of these data, anecdotal experiences in lay press and social media lead to misinformation and confusion for the public. In the revised submission, we more clearly state our knowledge gaps and implications of our findings.

Additional Requirements:

1. Please ensure that your manuscript meets PLOS ONE's style requirements, including those for file naming. The PLOS ONE style templates can be found at https://journals.plos.org/plosone/s/file?id=wjVg/PLOSOne_formatting_sample_main_body.pdf and https://journals.plos.org/plosone/s/file?id=ba62/PLOSOne_formatting_sample_title_authors_affiliations.pdf

   We have reviewed PLOS ONE's style requirements and have ensured that the resubmitted manuscript meets these guidelines

2. Please ensure that you have discussed whether all data were fully anonymized before you accessed them.

   All Personally Identifiable Information was removed prior to being accessed for analysis. Only the minimum data necessary for this analysis was accessed.

3. Please provide additional details regarding participant consent. In the ethics statement in the Methods and online submission information, please ensure that you have specified what type you obtained (for instance, written or verbal, and if verbal, how it was documented and witnessed). If your study included minors, state whether you obtained consent from parents or guardians. If the need for consent was waived by the ethics committee, please include this information.

   We have included this information in the revised manuscript. Thank you for this comment.

4. We note that the grant information you provided in the 'Funding Information' and 'Financial Disclosure' sections do not match.

   When you resubmit, please ensure that you provide the correct grant numbers for the awards you received for your study in the 'Funding Information' section.

   Funding information was solely input in the PLOS Editorial Manager submission system. All funding sources are not considered grants or awards.

5. We note that you have stated that you will provide repository information for your data at acceptance. Should your manuscript be accepted for publication, we will hold it until you provide the relevant accession numbers or DOIs necessary to access your data. If you wish to make changes to your Data Availability statement, please describe these changes in your cover letter and we will update your Data Availability statement to reflect the information you provide.

   The genomes referenced in our study are currently available on GISAID, with virus name "hCoV-19/USA/CA-UCSF-JDxxxx/2022".

6. Please review your reference list to ensure that it is complete and correct. If you have cited papers that have been retracted, please include the rationale for doing so in the manuscript text, or remove these references and replace them with relevant current references. Any changes to the reference list should be mentioned in the rebuttal letter that accompanies your revised manuscript. If you need to cite a

retracted article, indicate the article's retracted status in the References list and also include a citation and full reference for the retraction notice.

We have reviewed the references and can confirm they are complete and correct.
